# Latest Trends in Sustainable Polymeric Food Packaging Films

**DOI:** 10.3390/foods12010168

**Published:** 2022-12-29

**Authors:** Edilson G. S. Silva, Sara Cardoso, Ana F. Bettencourt, Isabel A. C. Ribeiro

**Affiliations:** Research Institute for Medicines (iMed.ULisboa), Faculty of Pharmacy, Universidade de Lisboa, Avenida Prof. Gama Pinto, 1649-003 Lisboa, Portugal

**Keywords:** food packaging, 3D printing, plastic, biopolymer, films

## Abstract

Food packaging is the best way to protect food while it moves along the entire supply chain to the consumer. However, conventional food packaging poses some problems related to food wastage and excessive plastic production. Considering this, the aim of this work was to examine recent findings related to bio-based alternative food packaging films by means of conventional methodologies and additive manufacturing technologies, such as 3D printing (3D-P), with potential to replace conventional petroleum-based food packaging. Based on the findings, progress in the development of bio-based packaging films, biopolymer-based feedstocks for 3D-P, and innovative food packaging materials produced by this technology was identified. However, the lack of studies suggests that 3D-P has not been well-explored in this field. Nonetheless, it is probable that in the future this technology will be more widely employed in the food packaging field, which could lead to a reduction in plastic production as well as safer food consumption.

## 1. Introduction

Nowadays, food packaging is fundamental to ensuring food distribution and protection around the world, especially when considering the solid growth of the population. Without packaging, food would easily spoil and the distribution of enormous quantities of food, raw and processed, to different areas around the globe would hardly be possible [1,2]. Among its functions, food packaging protects food from contamination and physical damage, maintains its freshness, improves its shelf-life, and gives relevant information about its contents [1,2]. Despite its effectiveness, conventional food packaging poses some concerns, such as food spoilage, since plastic itself has no effect on microorganism contamination, as well as excessive production of fossil-based plastic—this sector being one of those that employ this type of material most heavily, food packaging representing more than 40% of total plastic production [3]. Since fossil-based plastic is inherently non-renewable and non-biodegradable and its production has been massively increasing in the last seven decades (from 2 million tons in 1950 to 367 million tons in 2020) [3,4], new alternative materials for the manufacture of food packaging have been sought.

One emerging set of alternatives that have been studied as potential solutions to the above-mentioned problems are bio-based food packaging films functionalized with compounds of natural origin, since they are characterized by both biodegradability and renewability, in addition to active and/or intelligent functions [5,6,7]. In the production of these bio-based films, different methodologies may be employed depending on the purpose in question, the most common methods being solvent casting, layer-by-layer assembly, and extrusion. Moreover, additive manufacturing technologies, also known as “3D printing” have been, although scarcely, used in the production of bio-based films and other types of bio-based packaging. Considering the importance of food packaging, its current drawbacks, and the potentialities of 3D printing, the objective of this review is to explore progress in the production of bio-based films by conventional methodologies and additive manufacturing and to investigate how this technology can contribute for the development of bio-based sustainable primary food packaging. For this, the literature was examined to find recent research on developments and advances in bio-based and functional food packaging produced by conventional methodologies and by 3D printing, as well as advances in bio-based alternative feedstocks for 3D printing with potential application in the food packaging area. Furthermore, perspectives on and limitations of additive manufacturing applied in the production and development of bio-based primary food packaging are presented in order to try to understand why this innovative and powerful technology has been barely explored in this field.

## 2. Novel Materials for Food Packaging Films: Biopolymers and Additives

**Figure 1:**
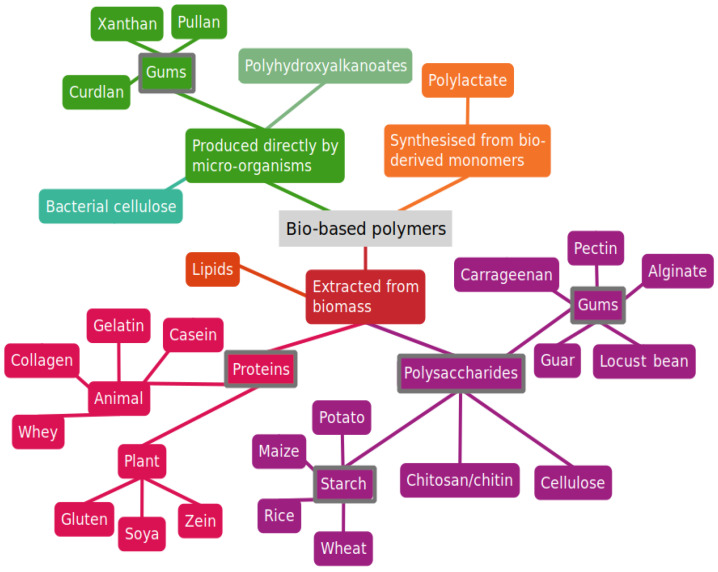
Bio-based polymers used in food packaging applications.

As alternatives to conventional plastic-based packaging, bio-based polymer films have been explored as potential candidates for the development of food packaging. Regarding the advantages over conventional petroleum-based food packaging, these bio-based films can decrease carbon dioxide levels, do not release dangerous substances into the environment, can be degraded by naturally occurring bacteria, can reduce the amount of waste generated, and are non-toxic [8,9]. Furthermore, the main materials used in the preparation of these films, that is, the biopolymers, are generally abundant in nature and can be derived from plenty of sources, namely, microorganisms, plants, animals, and food/agricultural wastes. Examples of biopolymers used in food packaging applications are summarized in Figure 1.

In addition to biopolymers for bio-based films, there is an increasing interest in compounds of natural origin, such as extracts and essential oils, as additives in the production of food packaging. When these natural compounds are incorporated into polymeric films, they can provide active properties, such as antioxidant, antimicrobial, and scavenging properties, that are crucial for food packaging, due to food deterioration and microbial contamination, which can produce off-flavors, lead to food spoilage, and cause food-borne diseases [7,10,11]. The active substances present in these compounds differ in composition and quantity and can be divided according to their structures and modes of action: phenols and phenolic acids, quinones, flavones and flavonoids, tannins, coumarins, terpenoids, alkaloids, and lectins and polypeptides [12]. In terms of antimicrobial activity, the modes of action of these substances can vary and include leakage from the bacterial cell, cell-shape damage, the destruction of cell walls, and alterations to membrane composition, among other mechanisms [12,13]. In addition to active properties, these compounds have also been used to provide extra and/or intelligent properties in films, such as sensitivity to pH changes; improved mechanical, thermal, optical, and barrier properties; and sensing abilities, among others [7]. In sustainable food packaging, the incorporation of these compounds can lead to the development of active materials that, in addition to being biodegradable/sustainable, can also increase food shelf life, reduce microbial contamination, and give information on food freshness [10]. Many natural compounds, such as natural extracts and essential oils, have been studied for the above-mentioned properties. Some examples of natural products that have been employed as sources of antioxidant and/or antibacterial compounds in bio-based food packaging include extracts of cranberries, cabbage, amaranth leaves, rosemary, cinnamon, broccoli, kale, and others, as presented [14,15,16,17,18,19].

## 3. Main Methods Used in the Production of Bio-Based Films

In the production of bio-based films, the methodology employed depends on the film application and on the objectives.

In brief, the fundamental step in processing any biopolymer film involves solubilizing and/or melting a biopolymer mixture, which is followed by the implementation of the desired technique [20]. Some of the most common methods for the production and application of bio-based films include the solvent casting method, layer-by-layer assembly, and coating and extrusion methods, which are briefly described in Table 1 [21,22]. Some of these methods are limited to lab scale, while others can be scaled up for industrial settings.

Solution casting is the simplest and most reported method in the literature on the production of bio-based films. The method consists of preparing a film-forming solution with an appropriate polymer–solvent concentration and casting it on a surface (e.g., a Petri dish, a glass plate) according to the desired thickness and uniformity of the films [22]. The drying conditions can vary and can merely involve drying the solution at room temperature or in an oven at a high temperature, with or without an auxiliary air system [23]. When the solvent evaporates, the film can be peeled off from the surface. Due to its simplicity and the mild conditions involved, solution casting is the method of choice in laboratory and scale-up experiments, but it is not practical at an industrial scale.

As an alternative, a film-forming solution can also be applied directly onto a food surface. This methodology, known as coating, can vary according to the nature of the food, the coating objective, and the film-solution viscosity [24,25].

Among the existing methods for coating are dipping, spraying, and brushing. The dipping method is based on the immersion of food in a film-forming solution and is suitable for viscous solutions. Spraying is based on the diffusion of film-forming-solution droplets through a spraying tool and is most suitable for less viscous solutions [24]. In brushing, the solution is applied on food using a brush or a similar tool. As in the other methods, the food is properly dried after the coating [25]. As it is a requirement that it be consumable, the composition of the coating must be of food grade; thus, the food can be eaten along with the coating.

The layer-by-layer assembly (LBL) approach is based on the deposition of alternating layers of oppositely charged compounds. This method allows for working with a variety of molecules, such as polysaccharides, nucleic acids, proteins, carbohydrates, synthetic polymers, and others. The formation of the multilayers can vary and be achieved by different methods, such as casting different layers of a solution on a surface, spray-coating layers directly onto the substrate’s surface, and dipping or immersing the substrate in different solutions [22,26]. Among the mentioned methods, dipping is advantageous in that it is not subject to geometrical restrictions, and, for that reason, it is one of the most frequently employed methods.

**Figure 2:**
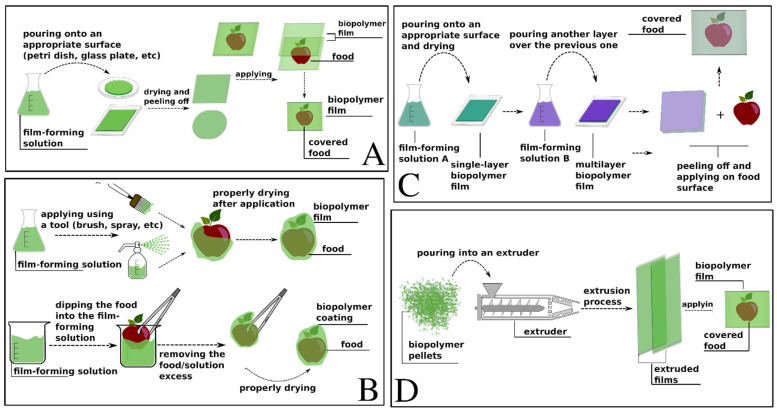
Conventional methods for the production of bio-based films. (**A**) Solution casting. (**B**) Coating. (**C**) Layer-by-layer assembly. (**D**) Extrusion.

In extrusion methods, polymers are mixed and extruded at high pressures and temperatures. In brief, a model of an extruder can be described as being composed of a hopper, which is where the raw materials enter the system; a barrel containing one or two rotating screws; and a die, where the polymer mixture leaves the equipment with the desired shape [27]. The rotating screws mix and transport the raw materials to the barrel, where the temperature is high and the polymer is melted, allowing for the incorporation of additives or other substances. An initial extrusion is conducted to produce pellets with the desired formulation, and these then pass through a second extrusion process to produce the films [22,28]. Figure 2 summarizes all four described methods.

Presently, there are many studies focusing on the use of different biopolymers functionalized with active compounds that can be obtained by different processes, as exemplified in Table 2. Nevertheless, there is still room for innovation. For example, an emerging methodology in the field of food packaging is additive manufacturing, which will be discussed in the following section.

## 4. Three-Dimensional Printing of Food Packaging and Films

Three-dimensional printing or additive manufacturing (AM) is a relatively new technology that has been revolutionizing a range of industries, research, and the overall manufacturing of new products because of its advantages, such as the reduction of manufacturing times, the possibility of producing complex shapes and parts, and the potential for innovation, and it has also been, although scarcely, used as a means to develop bio-based packaging materials. With this set of technologies, solid models are fabricated through the layer-by-layer deposition of raw materials, followed by their solidification, and it is possible to work with powder-based, liquid-, and solid-state feedstocks, depending on the chosen technology [48,49,50,51].

In general, the 3D printing process can be described as a sequence of steps, the first one being the generation of a computer-aided design of the desired object, followed by its conversion into a 3D object file, which will be read by the slicing software and built on the platform afterwards [49]. The principle of operation and type of 3D printing can vary depending on the application. In total, there are seven standardized processes (or techniques) that 3D printing is based on: binder jetting, directed energy deposition, material extrusion, material jetting, powder bed fusion, sheet lamination, and vat photopolymerization [50,52]. These processes differ in terms of the type and the state of the raw material used, in the degree of detail of the printed object, and in the fundamentals behind the printing process. According to Zhou et al., among the cited methods, material jetting, powder bed fusion, vat photopolymerization, and material extrusion are the most suitable techniques for printing 3D objects made of soft materials, such as polymers, and they will be briefly summarized below (see also Figure 2) [52].

### 4.1. Vat Photopolymerization

In vat photopolymerization, the 3D object is created by the solidification of a photopolymer resin when it is hit by light. In this process, a liquid photo-reactive polymer, which is contained in a vessel, is selectively cured by a UV light coming from a light source, forming a thin-layer cured polymer as a result [50]. The main 3D printing techniques that are based on this principle are stereolithography (SLA) and digital light processing (DLP), mainly differing in the sources of light [52]. The main advantages of vat photopolymerization are the high degree of accuracy and the smooth surface of the produced 3D object. Drawbacks include the need to use supports during the printing process and the inherent natures of the photopolymers employed as raw materials, such as their physical fragility and susceptibility to sunlight, which limit the range of applications of these products and make them less durable.

### 4.2. Material Jetting

Similar to vat photopolymerization, in material jetting, the object is formed by the solidification of a photo-sensible resin, but unlike the previous technique, this method is based on the deposition of tiny droplets of the photopolymer resin on the build platform, followed by their solidification by ultraviolet light [50]. This technique is regarded as the most accurate 3D printing technique and can produce objects with smooth surfaces and high degrees of detail. Analogous to vat photopolymerization, the main drawbacks of material jetting are related to the intrinsic properties of the raw materials, including the poor mechanical properties and the susceptibility to sunlight of the produced objects [49].

### 4.3. Powder Bed Fusion

The powder bed fusion process is based on the fusion of a powder-based material by a laser or an electron beam [50]. In this mode of operation, a thin layer of powder (e.g., a metal, ceramic, polymer, or composite) is distributed on the build platform and a laser automatically fuses layers of the material. This technology includes three printing techniques: electron beam melting (EBM), selective laser sintering (SLS), and selective heat sintering (SHS). The EBM and SHS techniques are mainly employed with metals, whereas SLS is employed for polymer materials (Redwood et al., 2017). The resolution of SLS is inversely proportional to the particle size, and it is preferable to use low-thermal-conductivity polymers as raw materials due to their stability in the fusing step [52]. Among its advantages, SLS produces objects with isotropic natures, making them stronger and more resistant than other printing technologies, such as FDM. In addition, SLS has a high degree of accuracy and, unlike vat photopolymerization, does not require extra supports to build objects [49].

### 4.4. Material Extrusion

Material extrusion is one of the most widely used 3D printing processes. The principles of this technique can be divided into two main groups based on whether the raw material is melted or not [53]. The technology based on the melting of a material is known as fused filament deposition modeling (FDM) and uses thermoplastics in the form of thin filaments as raw materials [50,52]. Another technology is direct ink writing (DIW), which is based on the extrusion of viscoelastic materials by means of pneumatic (air or pressure) or mechanical (screw- and piston-based) action, followed by the curing of the extruded material using photopolymerization or thermal processes [52,53]. Among the cited techniques, FDM is the most common method used in 3D printing. In FDM, a solid filament is extruded through a heated nozzle, melted, and selectively deposited on the build platform where it solidifies, forming a layer of the object. The advantages of this technique include the low costs of the materials and machines, the easy mode of operation, and a broad range of workable materials [48].

**Figure 3:**
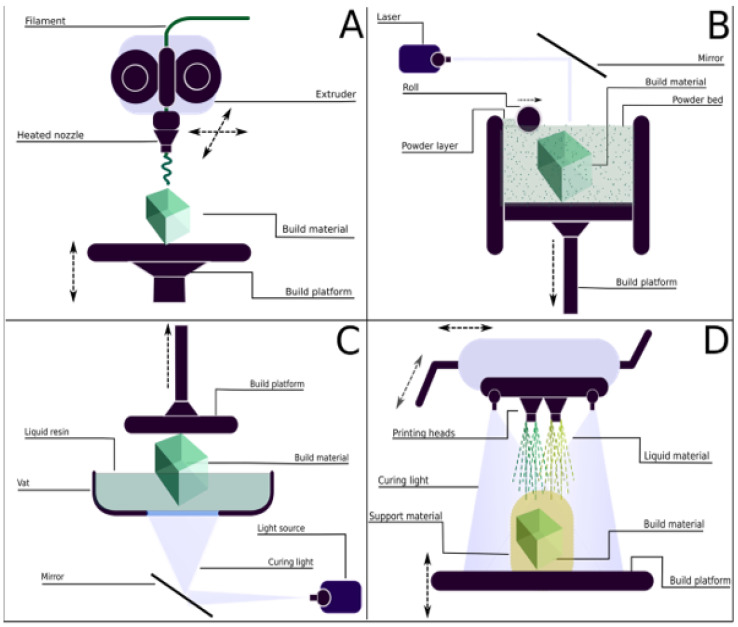
Three-dimensional printing techniques suitable for working with polymers. (**A**) Material extrusion. (**B**) Powder bed fusion. (**C**) Vat photopolymerization. (**D**) Material jetting.

The main limitation of extrusion-based 3D printing is related to the anisotropic nature of the produced objects, that is, the fragility of objects in one of their directions. The rheological and thermal properties of the material employed are also critical and depend on the nature of the extrusion process. Additionally, as is often the case with other AM technologies, it is likely that the final object will require some post-treatment to remove undesirable layer lines, the formation of which is inherent to the layer-by-layer building process [48,49]. Representations of the four described additive manufacturing techniques are presented in Figure 3.

## 5. Perspectives on AM in the Production of Bio-Based Films

As previously discussed, there are different additive manufacturing technologies that are compatible with polymers. However, not all of these technologies seem appropriate for applications in the food industry, especially for food processing and the production of primary food packaging. In the following section, research on the development of bio-based feedstocks for these technologies, as well as applications and/or potentialities in the food area regarding packaging production, will be addressed. Some of the studies presented may not be directly concerned with food packaging or related fields; however, most of the materials employed may be or have already been used in the production of films intended for food packaging.

### 5.1. Vat Photopolymerization and Material Jetting

One of the main driving factors of the research on bio-based photopolymers for 3D printing is the concern with sustainability issues, since most of the resins used in photo-based 3D printing technologies are derived from fossil resources [54]. Among sustainable alternatives to fossil-based materials are vegetable oils, lignin, chitosan, starch, and many others, which, after functionalization with photo-sensitive groups, such as acrylic or epoxy groups, can form solid shapes when cured by UV light [54].

Among the studies in this area is the work of Ding and coworkers [55], where they produced a high-biorenewable-content blend composed of natural phenolic acrylates, which was further evaluated as a photo-curable resin for SLA 3D printing. The acrylate compounds were synthesized from guaiacol, vanillyl alcohol, and eugenol and printed by a vat-photopolymerization-based 3D printer. The blends were then evaluated by real-time infrared and SEM, tensile strength, and thermal analyses. Based on the results, the researchers found that the produced blend had a high curing rate and a high glass-transition temperature, while the produced prototype showed good thermal and mechanical properties, although a few defects were observed on the printed surface.

Another interesting work in this area was conducted by Kim et al. [56], in which they produced a modified silk fibroin as a bioink for digital light processing intended for bioengineering applications. In their work, silk fibroin, a natural protein produced by silkworms, was functionalized with methacrylate groups, and its printability was evaluated by a DLP 3D printer. The mechanical, rheological, and water-uptake properties of the produced silk fibroin-based (Sil-MA) hydrogel were assessed, and, as a result, the research group found that the mechanical properties, such as compressive strain and compressive stress, increased as the concentration of Sil-MA increased, up to a 30% content of Sil-MA, at which the hydrogel prototype was able to support a 7 kg weight without being deformed after the weight’s removal.

Despite the advances in developing bio-based feedstocks for these AM technologies, it is unlikely that they will find application in food packaging fields. Firstly, resin-based AM technologies are reported to produce brittle and UV-sensitive objects, both characteristics inappropriate for food packaging films. In addition, many compounds used to prepare photo-sensitive resins, which are employed in these techniques, are considered toxic to some degree; therefore, due to safety and legal issues, it is unlikely that the produced objects will be suitable for contact with food.

### 5.2. Powder Bed Fusion

Unlike the above-mentioned techniques, powder bed fusion technology produces objects by means of the fusion of a powder material; therefore, a functionalization step with photo-sensitive groups is not required. Since this technique can employ less chemically modified materials as feedstocks, it is likely that this technology will find more applications with biopolymers in food-related fields than the previous two techniques. By contrast, the thermal properties and particle sizes of powder materials are of great relevance to this methodology. With respect to research on bio-based feedstocks for this technique, most of the published works consulted are concerned with regenerative medicine and similar fields.

In one of these studies, Dechet et al. [57] reported the production of spherical poly(L-lactide) particles for powder bed fusion using a sustainable method. The method, known as liquid–liquid phase separation, involves preparing a polymer solution with a poor solvent at a high temperature and subsequently cooling the solution so that the polymer precipitates and forms microspheres. In this work, triacetin, a green solvent derived from glycerol, was employed to solubilize the polymer. After producing the particles, by SEM analysis, the researchers found that, with increasing polymer concentration, the efficiency of the process increased, producing as a result more spherical particles with greater flowability. The specimens produced by poly(L-lactide) particles via powder bed fusion 3D printing showed good layer adhesion and good mechanical properties, comparable to those produced by the FDM process.

In another recent work, Gayer et al. [58] produced a solvent-free biodegradable PLA/calcium carbonate composite intended for bone-tissue engineering applications. The powder was prepared by processing a mixture of the two compounds in an impact mill, followed by a sieving step to obtain a narrow range of particle sizes. At the end of these processes, four powder mixtures, with calcium carbonate contents ranging from 22% to 27%, were obtained and characterized. The printability of the composite powders was assessed using an SLS 3D printer, and the obtained specimens were evaluated by mechanical strength, cell viability, and porosity assays. The results showed that the composite powder with 23% calcium carbonate content had the best processability, good mechanical strength, low melt viscosity, and small particle size, in addition to good cell compatibility.

In another interesting work using biopolymers and powder bed fusion, Diermann and coworkers [59] produced and evaluated scaffolds made of poly(3-hydroxybutyrate-co-3hydroxyvalerate) (PHBV) and Åkermanite, a sorosilicate mineral, as a filler, in vitro. The scaffold was intended for tissue engineering, taking advantage of PHBV properties, such as slow degradation and compatibility with the components of human blood [60]. For the preparation of the composite powder, PHBV powder was sieved to obtain a narrow particle size distribution and some of the as-received Åkermanite powder was ball-milled to obtain particles at micro- and nanoscales. Both powders were obtained commercially. After these steps, the powders were blended in a mixer for 8 h and sintered in an SLS machine to produce four scaffolds with different PHBV/Åkermanite ratios and different particle sizes. As demonstrated by the authors, the Åkermanite particles were well dispersed throughout the PHBV matrix, and the scaffold with microparticles had the best mechanical performance over the Åkermanite nanoparticles. Additionally, the incorporation of Åkermanite into the blend improved the water uptake of the scaffold—an important property for the intended application [59].

Although powder bed fusion seems more promising for working with bio-based polymers without further chemical modification, in contrast to vat photopolymerization and material jetting, this technique only works with solid-state materials, limiting its versatility in the production of films. In fact, no studies on the production of bio-based films using powder bed fusion were found.

### 5.3. Material Extrusion

Among the AM technologies studied, material extrusion seems to be the most appropriate for developing bio-based films and other materials for food packaging applications using either filaments or gels. Despite the fact that powder bed fusion technologies use polymers as feedstocks, no studies on the production of films using these materials were found. Other technologies discussed herein, such as vat photopolymerization and material jetting, seem not to be suitable for the production of bio-based food packaging due to the use of resins as their main materials, which are often non-compatible with food safety. Additionally, the objects produced with these technologies are known for having characteristics undesirable in films, such as brittleness and sensitivity to UV light. Extrusion-based technologies have a broader range of workable materials in comparison with resin-based AM technologies. Additionally, unlike powder bed fusion, extrusion-based 3D printers allow for working with biopolymers in solid (FDM) and gel–liquid (DIW) states, making them more versatile tools for working with bio-based polymers than the other AM technologies discussed herein. Considering this, some advances in the production of feedstocks for extrusion-based technologies using biopolymers with potential for application in food packaging, as well as advances in the production of films and food packaging using these materials, will be presented below. Among the biopolymers that can be used to produce packaging films by material extrusion techniques are lactic acid-based polymers, lignin, alginate, chitosan, starch, gums, cellulose and its derivatives, whey, and many others, some of which will be discussed below.

In one of these studies, Domínguez-Robles et al. developed a lignin/lactic acid-based filament with antioxidant properties intended for fused filament deposition modeling (FDM) [61]. The produced filament was extruded by a 3D printer, showing good mechanical properties and stability, keeping its integrity even after being immersed in phosphate-buffered saline solution for 30 days. The researchers were also able to successfully incorporate an antibiotic into the filament using a hot-melt extrusion process, demonstrating the possibility of incorporating multiple active compounds into the filament’s composition by the methodology employed.

Another bio-based filament for 3D printing was developed by Umerah and coworkers [62]. The filament was produced using a blend of coconut shell powder, polylactic acid, and a starch-based bioplastic. To produce the filament, coconut shell powder was immersed and subsequently precipitated in a solution containing the polymers. After being filtered, the precipitate was turned into a powder and extruded in the form of a filament. The produced filament was shown to have improved thermal and mechanical properties compared to the bioplastic per se, which was attributed to the coconut shell powder addition. The eco-friendly aspect of the composite, along with its non-toxicity, makes it a potential raw material suitable for food packaging applications.

In another interesting study with biopolymers, Hafezi et al. produced several chitosan-based films incorporating genipin—a fruit-derived compound with antibacterial properties [63]. For the film production, an appropriate gel using low-molecular chitosan and genipin as a crosslinker was prepared and further extruded by a 3D printer. After being extruded, the films were thermally cured in an oven and properly characterized. The researchers were also able to incorporate an organic compound into the films’ composition as a model drug. The films with the model drug incorporated into them were further evaluated in a drug-release assay, showing appropriate release rates for the intended application (wound healing) and demonstrating the possibility of incorporating active substances into the films’ matrices.

In one of the few studies found concerning the use of 3D printing and bio-based polymers in food packaging applications, Li et al. developed a double-composite intelligent film intended for monitoring and extending meat shelf life [64]. The film, which was chitosan-based, consisted of two layers, one prepared with lemongrass essential oils, the other with mulberry anthocyanin in its composition, both encapsulated by a starch-based film. For the production of the films, a chitosan solution was prepared with the active components and extruded by a 3D printer, followed by its curing at a controlled temperature. Starch films were also prepared and heat-sealed onto the active films. The final films had antioxidant and antibacterial properties due to the lemongrass essential oil presence and the ability to change color according to the pH of the medium in which they were placed due the pH-responsiveness of anthocyanin. The latter was further evaluated in the monitoring of fresh-meat spoilage, where the film successfully responded to changes in pH, changing in color from a reddish tone (at pH 2–6) to a blueish one (at pH 7–12). By an antibacterial assay, the researchers found that the addition of anthocyanin to the films had a bacteriostatic effect toward *E. coli*, in addition to the antibacterial effect provided by the lemongrass essential oil, which was effective in inhibiting both *E. coli* and *S. aureus*. By a release-rate assay, the researchers also found that the release rates of the active compounds supplied by the essential oil increased with increasing pH, suggesting that the active properties could be even more effective in increasing food shelf life. Overall, the produced films showed great promise as innovative primary food packaging materials.

In the work of Wang et al., a chitosan-based active film was produced by the solvent casting method and 3D-printed after the appropriate formulation was found [65]. In the film production, the researchers used chitosan as the film-forming substance, tea polyphenols as a source of active compounds, and nanotubes of halloysite—a naturally occurring aluminosilicate—as fillers to improve some properties of the films and control the release of active compounds. After evaluating the films made by the solution casting method, the researchers found the best formulation to produce the bio-based ink for an extrusion-based 3D printer. The ink was successfully extruded, producing thin smooth films with both good antioxidant and antibacterial activities against a variety of bacteria, including *E. coli* and *S. aureus*. Furthermore, the halloysite addition improved the films’ mechanical properties, with no further reduction in printability. In a further work, the researchers employed a similar formulation to produce a bio-based food packaging container by means of 3D printing [66]. The container was evaluated with respect to the preservation of fresh blueberries and was able to maintain fruit freshness for a longer period in comparison with a blank control and a pure chitosan container, showing less loss of weight, firmness, and ascorbic acid contents [66].

In the work of Biswas and coworkers, another active food packaging film was formulated and 3D-printed. For this, they synthesized and incorporated silica–carbon–silver nanoparticles into a biodegradable polymer known by its brand name “Ecoflex” [67]. The objective of using nanoparticles in this work was to add antibacterial properties to the films as well as to improve the films’ mechanical and thermal properties. The nanoparticles were synthesized using rice husks, an agro-industrial waste, and silver nitrate by means of thermal treatment and a ball-milling process. After being synthesized, the nanoparticles were incorporated into a film-forming solution containing the polymer, and the resultant solution was printed by an extrusion-based 3D printer. The researchers evaluated the antibacterial activity of the films against *Salmonella enteritidis* and found that the films possessed a bacteriostatic effect which was able to effectively inhibit the studied bacteria by contact. In order to evaluate the release of the films’ nanocomponents, the team conducted a silver-release test, in which the films were immersed in water for one week. No trace of silver was found in the studied period, suggesting that the produced films have the potential to be used as food packaging materials [67].

Other work worth mentioning in the food packaging field was performed by Ahmed et al., in which they developed a composite gelatin-based film with zinc oxide and clove essential oil [68]. In the film’s formulation, zinc oxide (considered a Generally Recognized as Safe (GRAS) substance by the FDA) was employed to improve the film’s properties and add inhibitory activity, and clove essential oil was used to add antibacterial and antioxidant properties. According to the authors, the presence of both active compounds would have a symbiotic effect on the film’s properties: while the addition of clove essential oil would negatively affect some mechanical properties, the zinc oxide, which does not possess the same efficiency in terms of active properties, would act as a filler and improve the film’s overall properties. After finding an appropriate film formulation by the solvent casting method, the researchers produced a semi-solid paste by hot-melt extrusion which was further extruded by a 3D printer to produce the bio-based films. The produced films showed improved mechanical properties in comparison with the control (pure gelatin), besides complete antibacterial activity towards both *L. monocytogenes* and *Salmonella typhimuriums*. Additionally, as suggested by the authors, the use of hot-melt extrusion in conjunction with 3D printing has the potential to optimize film production by means of this technology, which is beneficial, since 3D printing technologies are generally considered slow methods of production.

Another interesting work involving 3D printing in the food packaging field was conducted by Zhou et al., in which a bio-based active food packaging container was produced [69]. The container was produced by means of coaxial 3D printing, where a core–shell structure made of cellulose nanofibers incorporated with blueberry anthocyanin was loaded with chitosan and 1-methylcyclopropene (1-MCP)—a compound used for slowing the ripening of fruit. The idea behind the coaxial structure was to effectively control the release of the active components. For this, a cellulose-based ink was prepared using anthocyanin and both sodium alginate and K-carrageenan gums in order to improve the ink viscoelastic properties. This ink was subsequently printed, along with chitosan and 1-MCP in its core, and the resultant object was appropriately cured. By a pH evaluation, the researchers confirmed the pH sensibility of the container, and the release behavior of 1-MCP was evaluated by gas chromatography. In a further assay, the labels, as the authors refer to the printed containers, were evaluated for the monitoring and extension of the freshness of litchis and were found to successfully prolong fruit shelf life for six days, in addition to visually indicating changes in the litchis’ freshness.

Besides the production of new 3D printing feedstocks using bio-based polymers, in the literature there are also reports concerning the reuse or recycling of materials with similar purposes. One interesting work on the recycling of materials for 3D printing was conducted by Cisneros-López et al., in which they evaluated the production of biocomposites for material-extrusion-based 3D printers based on recycled polylactic acid [70]. The blends that the researchers produced were made with 30% recycled polylactic acid in a matrix of virgin polylactic acid, along with microcrystalline cellulose and an epoxy-based chain extender. The blend was extruded by a twin-screw extrusion process to produce the filaments, and the latter were printed using a FDM 3D printer. The researchers compared the performance of the 3D-printed objects with an injection-molding process utilizing the same blend and found that the 3D-printed objects had lower viscosities compared to the ones produced by the injection-molding process. Furthermore, the addition of micro-crystalline cellulose and the epoxy-based chain had a positive effect on the blend, improving both the mechanical and thermal properties of the produced filament [70]. A summary of research on 3D printing with biopolymers relevant to the food industry can be found in Table 3.

## 6. Limitations of 3D Printing in the Production of Films

Undoubtedly, additive manufacturing technology has great potential in the food packaging field; however, research in this area is still very limited. Most studies on AM technology and biopolymers are concerned with medical, textile, and pharmaceutical applications and the “tailor-made” characteristics of 3D printing, along with the biodegradability, abundance, low cost, and biocompatibility of the biopolymers used which make them suitable for the fabrication of biodegradable scaffolds, tissue and organ engineering, drug delivery systems, and innovative textile products [72,73,74,75]. With regard to the food industry, most research on AM and biopolymers aims at the production of customized food, as discussed in the previous section. In the few studies found on the development of films or materials for food packaging, AM technology proved to be very useful, allowing for the production of innovative and functional bio-based packages with controlled release of active substances.

Considering the lack of research on foodstuff packaging and the fact that additive manufacturing is a relatively new technology that has been on the market for no more than a couple of decades, it is obvious that more studies on AM focused on the development of food packaging are needed. Additionally, in order to explore the potentiality of 3D printing in the food packaging area, some challenges must be overcome. To begin with, one must bear in mind that, given the current state of AM technology, its uses are confined to the development and research of bio-based packaging films rather than their industrial-scale production. This is due to the fact that, despite being faster than conventional methods for producing complex objects, AM is still considered a slow process and can take from hours to days to produce an object, depending on the object’s complexity [76]. In addition to the above, depending on the printer specifications and the final purpose, AM technology can be very costly and can include the costs for 3D printer machines, materials, and post-processing [51]. Adding these two shortcomings together, it is unlikely that large-scale production of 3D-printed objects will be possible without further modifications or improvements.

Another challenge in the AM technology field is presented by the physicochemical properties of the biopolymers used, such as the minimum requirements for the biopolymers to be processed by 3D printing technologies, as well as the properties that are desired in the final products after processing. For instance, in FDM, which is by far the most intensively explored AM technology, one requirement is that the biopolymer should be thermally stable and melt-processable, this being a challenge for most biopolymers, since they generally have lower thermal stability, heat-flowability, and a narrower range of workable temperatures in comparison with their petroleum-based counterparts [77]. In the preparation of feedstocks for AM, solubility is another key property. Some biopolymers, such as cellulose, have inherently low solubilities in common solvents, making it difficult for them to be processed by AM technologies. In the case of cellulose, strategies to properly dissolve and regenerate it have been employed using ionic liquids and other non-standard solvents, but it still poses a challenge for AM processing [78,79]. In contrast, the highly hydrophilic natures of some biopolymers may compromise their final applications, especially if they are to be used in packaging films, where good barrier properties are essential to the packages’ providing effective protection. These and other drawbacks, such as thermal instability, brittleness, stiffness, low barrier properties, and vulnerability to degradation, need to be improved in order for these alternative materials to be successfully used in food packaging applications [8]. Regarding the production of intelligent and active films, another interesting issue is the evaluation of as-produced films in order to identify possible alterations to the films’ active properties after processing by AM technologies.

Some strategies to overcome these challenges include the study of appropriate formulations and/or functionalization of the biopolymers aiming at improving their properties for better AM processability. Adaptations of AM technology may also be necessary to improve efficiency and performance in the packaging field by means of bio-based polymers, including greater compatibility with alternative feedstocks, better processing speed, and general optimizations of the overall technology to reduce costs. Nonetheless, the precision, automation, and versatility of AM technology can clearly contribute to significant advances in the production and development of bio-based packaging films.

## 7. Conclusions and Future Perspectives

**Figure 4:**
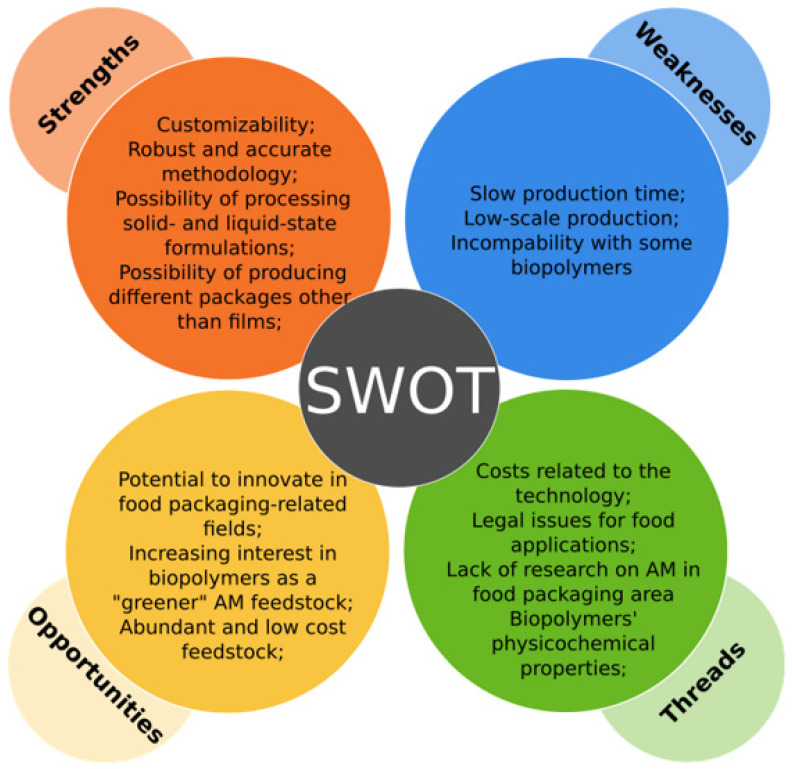
SWOT analysis of 3D printing as a tool for the production of bio-based films for food packaging applications.

The aims of this work were to explore the progress in developing bio-based alternatives to conventional plastic packaging as well as to examine how additive manufacturing technologies can contribute to the development of bio-based food packaging films. To attain these goals, the literature on the production and development of biopolymer-based films and primary food packaging by conventional methods and by means of AM, as well as alternative feedstocks for AM relevant to food packaging development, was reviewed and discussed. Based on the information extracted from the studies, bio-based films and food packages developed by means of AM technologies, as well as promising feedstocks for these technologies, were identified. Among the employed biopolymers, we highlight chitosan, polylactic acid, cellulose and its derivatives, starch, gums, and polyhydroxyalkanoates—all of which can be used, individually or in blends, in the production of sustainable films. Additionally, the use of active substances of natural origin was also found in the development of active bio-based packaging. Along with the biopolymers, these compounds allow for the development of packaging formulations that are not only biodegradable and sustainable, but also possess active and intelligent properties, such as antibacterial activity, antioxidant activity, sensitivity to pH changes, and resistance to ultraviolet radiation. From the findings, it was concluded that, despite the promising works directly related to the development of bio-based food packaging by AM, this technology has not been well explored in this field. Most of the research concerning the development of bio-based feedstocks for AM is aimed at biomedical, pharmaceutical, and textile fields, where the precision, automation, and the ability to build complex shapes and tailor-made objects, along with the biodegradability, biocompatibility, and the abundance of biopolymers in AM, promote advances in the development of tissues, organs, scaffolds, drug delivery systems, and smart and innovative textile products, among other tailor-made objects in these areas. At present, in the food industry, AM applications are mainly directed at the production and development of customized food. A brief overall SWOT analysis of the potential of 3D printing as a tool in the production of biopolymer-based films for food packaging applications is presented in Figure 4.

The factors that contribute to the lack of research on food packaging films produced by AM might include the high costs associated with AM technologies, the incompatibility of biopolymers with 3D printing, the relatively slow production methods, the scaling-up difficulties, and the need to develop biopolymer blends/formulations with not only good printability but also the properties that meet the necessary criteria for food packaging materials.

Despite the lack of studies on the production of bio-based materials for food packaging applications by AM, this technology still seems very promising in this field. Furthermore, it is very likely that this area will benefit from the advances related to AM and biopolymers in other fields. As the feedstocks and the technology employed are the same, adaptations in terms of better compatibility/processability in AM regarding biopolymers of relevance to these various fields would probably benefit the production of bio-based food packaging by means of this technology as well. In this respect, it is very likely that, as AM is gradually better adapted for the processing of biopolymers and these materials are increasingly explored in relation to this technology, the potential for 3D printing as a more effective and less limited tool in the production and development of biopolymer-based primary food packaging will increase.

## Figures and Tables

**Table 1:** Main methods for preparation of biopolymer-based films (Kumar et al., 2020; Wang et al., 2018).

Method	Main Characteristics
Solution casting	The film-forming solution is cast on a surface (e.g., a Petri dish), appropriately dried, and the formed film is peeled off; It is the simplest method for film preparation; The conditions used are relatively mild; It is time-consuming; It is limited to lab scale;
Coating	The film-forming solution is directly applied onto the food by means of dipping, spraying, or brushing and dried afterwards in appropriate conditions; Often applied on fresh food; Materials must be of food grade if the coating is meant to be eaten; Some applications in the food industry (mostly wax coatings);
Layer-by-layer assembly	Based on the deposition of alternating layers; Deposition can be achieved either by submersion in or spraying the film-forming solutions on the food; Potential for industrial applications, though currently it is mostly limited to lab scale;
Extrusion	The mixture containing the biopolymer is poured into an extruder system, which produces a uniform film at the end of the process; Faster and less energy-demanding than the solution casting method; Produces films with superior mechanical and thermal properties; Conditions may be aggressive for biopolymers; Can be scaled up for industrial settings;

**Table 2:** Advances in the development of bio-based food packaging films.

Methodology	Aim	Used Biopolymers	Other Components	Properties	Ref.
Casting	Effect of chitosan molecular weight on film performance	Chitosan	-	Improved preservation abilities; Improved performance with high chitosan weights/contents	[29]
	Influence of chitosan molecular weight on the film properties	Chitosan + bacterial cellulose	Curcumin	Improved performance with high chitosan weights/contents	[30]
	Develop a multifunctional food packaging film	Chitosan	Alizarin	pH-responsive film (4–10 range); Improved thermal stability, hydrophobicity, and UV-blocking properties	[31]
	Effect of a starch source on the performance of edible starch-based films	Starch (tapioca, rice, potato, and wheat)	-	Tapioca, potato, and rice starch had better mechanical strength and less color difference	[32]
	Production of biodegradable cellulose/alginate films	Cellulose, alginate, and carrageenan	-	Films showed weight loss of up to 50% after 60 days buried in soil; Activity against *E. coli*, *Pseudomonas syringae,* and *S. aureus* strains	[33]
	Develop UV absorbent films	Polylactic acid	Grape syrup	High UV absorption property	[34]
	Develop functional bio-hydrogel films for food packaging	Alginate, agar, and collagen	Grapefruit seed extract Silver NPs	Improved mechanical properties; High UV screening; Strong antimicrobial activity: prevents greening of fresh potatoes	[35]
	Study the influence of nano-SiO_2_ concentration on the properties of the films	Agar + alginate	Silicon oxide NPs	Improved mechanical properties	[36]
	Evaluated the effect of fatty acid chain length on the properties of edible films	Basil seed gum-based	Caprylic, lauric, and palmitic acids	Improved barrier properties; Improved mechanical properties (lauric and caprylic acids)	[37]
	Develop intelligent films for food packaging	Gellan gum and soy protein	*Clitoria ternatea* extract	pH-responsive (3–11 range); Bacteriostatic activity	[38]
	Evaluate the influence of the concentration of the extract on the properties of films	Starch	Red cabbage extract	Antioxidant activity; Significantly increased food shelf life (meat)	[17]
	Develop a composite film and evaluate its activity as primary food packaging for fresh poultry	Chitosan	Rosemary essential oil and montmo-rillonite	Antioxidant and antimicrobial activity; Improved barrier properties; Films were able to retard lipid peroxidation (poultry)	[19]
	Develop a film with mesoporous silica NPs loaded with clove essential oil	Polylactic acid	Clove essential oil and silica NPs	Antimicrobial activity; Controlled the release of the active compound	[16]
Coating and solvent casting	Develop a film-forming formulation and compare the effects of different applications (coating, wrapping, and direct application of active compounds) on food	Alginate and cellulose	*Ziziphora* essential oil, apple peel extract, and zinc oxide nanoparticles	Coating showed the lowest bacterial population and best sensory attributes among the studied methodologies	[39]
	Investigate the potential of cranberry extract as an antibiofilm additive for a chitosan-based film	Chitosan	Cranberry extract	Antioxidant and antimicrobial activities	[18]
Coating	Develop a superhydrophobic food-grade coating	-	Candelilla and rice bran waxes	Highly hydrophobic coating; Excellent coating resistance to physical damage	[40]
LBL and coating	Compare the effects of coatings by means of LBL and standard coating	Chitosan; Cellulose	-	Single-layer and LBL coatings had positive effects on strawberry conservation; LBL coating showed better performance at reducing firmness and volatile compound loss	[41]
LBL	Development of a bilayered chitosan/FucoPo film	Chitosan and FucoPol	-	Improved gas barrier towards O_2_ and CO_2_ in comparison with monolayer film	[42]
	Prepare and characterize an antibacterial film	Chitosan and modified polyethylene	Hyaluronic acid	Excellent antibacterial activity; Improved degradability	[43]
LBL and solution casting	Evaluate the effects of preparation methods on the properties of the films	Chitosan and alginate	Ferulic acid	Crosslinked LBL films showed better results with improved mechanical, thermal, optical, and barrier properties	[44]
Extrusion	Study the effect of nanofillers on extruded films	Chitosan and starch	Nanoclay and bamboo fibers	Improved mechanical, thermal, and barrier properties	[45]
	Investigate the effects of nanoclay contents and pH levels on the properties of the films	Soy protein	Nanoclay	Nanoclay addition improved mechanical and rheological properties; pH changes demonstrated to have positive effects on film properties	[46]
	Evaluated the potential of the extrusion process and wax source on edible film properties	Rennet casein	Potassium sorbate; bee, candelilla, and carnauba waxes;	Beeswax had the best performance in terms of improving mechanical properties and hydrophobicity; Wax incorporation allowed a controlled release of potassium sorbate	[47]
	Develop a composite film and evaluate its activity as primary food packaging for fresh minced meat	Starch	Sappan and cinnamon herbal extracts	Improved barrier properties; Reduced microbial counts; Preservation of redness of packaged meat	[15]

**Table 3:** Studies on 3D printing with biopolymers of relevance to the food industry.

AM Technology	Polymer/Active Compounds and Fillers	Proposed Application	Properties	Ref.
Vat photo-polymeriza -tion	Guaiacol, vanillyl alcohol, and eugenol (acrylates)	Sustainable 3D-printing feedstock formulation	Good thermal and mechanical properties	[55]
	Silk fibroin (acrylate)	3D bioprinting in tissue engineering applications	Improved mechanical properties	[56]
Powder bed fusion	Polylactide	Sustainable 3D-printing feedstock formulation	Good layer adhesion and good mechanical properties	[57]
	Polylactic acid/calcium carbonate	Tissue engineering	Good processability, mechanical properties, low melt viscosity, and small particle size	[58]
	Hard keratin	Sustainable 3D-printing feedstock formulation	Weaker mechanical properties; Successful keratin incorporation/proce- ssing	[71]
	Polyhydroxyalkanoate/akermanite	Tissue engineering	Improved water-uptake properties	[59]
Material extrusion	Lignin and polylactic acid	Wound healing	Good mechanical properties and stability; Successful incorporation of an antibiotic	[61]
	Polylactic acid and starch/coconut shell	Sustainable 3D-printing feedstock formulation	Improved thermal and mechanical properties	[62]
	Chitosan/genipin	Wound healing	Good release rate of the active compound	[63]
	Chitosan and starch/lemongrass essential oil and mulberry anthocyanin	Food packaging	Color-changing properties; Antibacterial effect	[64]
	Chitosan/tea polyphenols and halloysite nanotubes	Food packaging	Good antioxidant and antibacterial activity; Improved mechanical properties	[65]
	Bio-based plastic “Ecoflex”/silica–carbon–silver nanoparticles	Food packaging	Bacteriostatic effect	[67]
	Gelatin/zinc oxide and clove essential oil	Food packaging	Improved mechanical properties and antibacterial activity	[68]
	Chitosan and cellulose/blueberry anthocyanin and methylcyclopropene	Food packaging	Color changing properties and preservation ability	[69]
	Polylactic acid (virgin and recycled)	Sustainable 3D-printing feedstock formulation	Improved both mechanical and thermal properties	[70]
